# Aberrations in Lipid Expression and Metabolism in Psoriasis

**DOI:** 10.3390/ijms22126561

**Published:** 2021-06-18

**Authors:** Julia Nowowiejska, Anna Baran, Iwona Flisiak

**Affiliations:** Department of Dermatology and Venereology, Medical University of Bialystok, Zurawia 14 St, 15-540 Bialystok, Poland; julia.nowowiejska@umb.edu.pl (J.N.); iwona.flisiak@umb.edu.pl (I.F.)

**Keywords:** psoriasis, lipids, cholesterol, triglycerides, fatty acids, lipoprotein

## Abstract

Psoriasis (PSO) is a common skin disease that affects about 1%–3% of the general population. It is a great medical, social and economic burden since PSO is associated with many comorbidities, of which the most common are cardiometabolic disorders. Psoriatic patients suffer more frequently from obesity, dyslipidemia, atherosclerosis, and nonalcoholic fatty liver disease. Research shows that lipid expression and metabolism disorders are present more often in such patients. This review focuses on a variety of aberrations in lipids in the skin, blood, and adipose tissue in psoriatic patients and their multifactorial impact on the pathogenesis of psoriasis.

## 1. Introduction

Psoriasis (PSO) is a common skin disease that affects about 1%–3% of the general population [1]. The most frequent variant is plaque PSO [2]. It manifests as erythematous-papular lesions with superficial scaling, which are usually located on the extensive surface of elbows, knees, and the lumbosacral area. Moreover, scalp and nails may be involved [2,3]. PSO has been proved to be accompanied by multiple comorbidities [4]. It is estimated that about 30% of patients will develop psoriatic arthritis [3]. Other disorders occurring more frequently in psoriatic patients are inflammatory bowel diseases, neoplasms, and neurodegenerative and sleep disorders [4,5]. Nevertheless, the most thoroughly investigated and closest relationship of PSO is probably that with metabolic syndrome (MS) and other cardiometabolic diseases (CMDs) [3,6].

**Figure 1:**
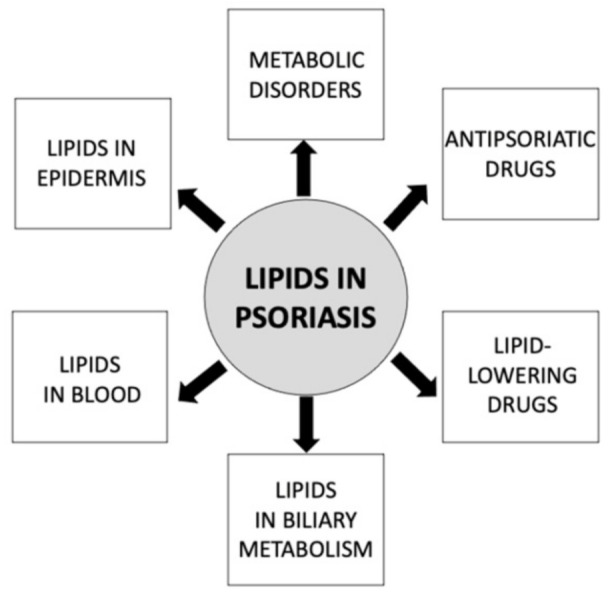
Multiple associations of lipids with different aspects of psoriasis.

Lipids are a family of various organic substances which play diverse roles in the human organism. They are an important component of the cellular membrane and take part in different biological processes, including cell proliferation and apoptosis, vessel formation, inflammatory conditions, or immune response [7]. The field of lipidomics analyzes the structure, functions, and interactions of lipids, in particular, the human body element, and may be closely applied in the investigation of psoriasis (Figure 1) [7].

## 2. Associations between Psoriasis and Metabolic Disorders

Metabolic syndrome (MS) is a disorder that can be diagnosed if a patient meets three of the following five criteria: increased waist circumference of ≥80 cm in females and ≥94 cm in males; triglycerides serum concentration of >150 mg/dL or treatment of hyperglyceridemia; HDL-C (high-density lipoprotein) serum concentration of <40 mg/dL in males and <50 mg/dL in females or treatment of this disorder; fasting blood glucose of ≥100 mg/dL or treatment of type 2 diabetes mellitus; systolic blood pressure of ≥130 mm Hg or diastolic blood pressure of ≥85 mm or treatment of hypertension [8]. MS is also associated with atherosclerosis and liver disorders, particularly nonalcoholic fatty liver disease (NAFLD) [8].

MS is estimated to affect about 20% to 50% of patients with PSO [8]. It may lead to an increased risk of cardiovascular complications [9], and it has been proved that psoriatic patients live on average five years less than people without this condition [10].

### 2.1. Links between Psoriasis and Obesity

Adipose tissue is a type of connective tissue which consists of cells, mainly adipocytes, and the extracellular matrix [11]. Obesity is defined as body mass index (BMI) over 30 kg/m^2^ [12]. As described above, its visceral type is the basic criterion of MS [12]. A meta-analysis conducted by Armstrong et al. revealed that pooled OR for all obese psoriatic patients was 1.66 (95% CI 1.46–1.89) [12], which means that the adipose tissue content in psoriatic patients is increased. There is a polymorphism in the *FTO* rs9930506 gene, which encodes 2-oxoglutarate-dependent nucleic acid demethylase and is associated with higher BMI and greater predisposition for obesity. At the same time, in subjects with such polymorphism, more severe psoriatic lesions are observed [8,13]. Moreover, the risk of PSO may be increased in obese people who possess the HLA Cw6 antigen [8,14]. Recently, a bidirectional relationship between PSO and obesity has been highlighted, namely PSO predisposing to obesity, and obesity enhancing psoriasis [15].

Adipokines are biologically active substances secreted mainly by the adipose tissue; however, cells other than adipocytes may also take part in their production [16]. They have properties of cytokines, hormones, enzymes, or growth factors [17]. To date, the role and concentration of different adipokines in PSO have been widely investigated, including by our team [18]. Depending on their properties, the concentration of some adipokines in serum and psoriatic tissues are elevated or decreased and correlate or do not correlate with BMI or PASI (Psoriasis Activity and Severity Index) [16].

### 2.2. Links between Psoriasis and Atherosclerosis

Atherosclerosis is a metabolic disorder characterized by the presence of atherosclerotic plaques in the arterial walls. A fatty streak is the first visible lesion suggesting atherosclerosis. It is an area of foam cells loaded with lipids in the intima of arteries. Atherosclerotic plaque, which develops from the fatty streak, comprises a core with amorphous material, lipids, and necrotic cells, and a capsule surrounding this central content, which may contain smooth muscle and inflammatory cells and connective tissue matrix [19]. Risk factors of atherosclerosis progression are, for instance, arterial hypertension, diabetes mellitus (DM), hypercholesterolemia, and smoking, conditions that occur more often in psoriatic patients. The plaque grows eccentrically over time, containing more lipids, including cholesterol esters, and becomes softer and more prone to disruption [19]. In addition, an exceptional observation is that atherosclerotic plaque has similarities to psoriatic plaques. Both plaques develop due to chronic inflammatory conditions and are linked to similar immune processes with particular cytokines (especially IL-17) and cells (T lymphocytes), as well as the involvement of thrombotic agents [20]. The process of forming a psoriatic plaque is also analogous to the formation of an atherosclerotic plaque.

### 2.3. Links between Psoriasis and Nonalcoholic Fatty Liver Disease

NAFLD is a condition characterized by fat accumulation in the liver. It is associated with neither alcohol abuse nor drug side effects [21]. NAFLD is closely related to MS, similar to psoriasis. It is estimated that 30% of the total population and over 50% of psoriatic patients suffer from NAFLD [22]. Significant fat deposition, especially visceral obesity, contributes to insulin resistance and has the advantage of proinflammatory cytokines, which favor NAFLD development. As obesity occurs more frequently in psoriatic patients, and because PSO and metabolic disorders have similar signaling pathways through IL-17 and TNFα, such patients are more prone to NAFLD [21,23]. Research suggests that the state of chronic inflammation in PSO may contribute to progression from healthy liver to NAFLD [23]; therefore, a concept of a ‘hepato-dermal axis’ has been introduced [24]. A reverse relationship has also been observed since proinflammatory agents are released from abnormally functioning liver tissue and lead to exacerbation of skin lesions [24].

## 3. Aberrations in Lipid Expression in Psoriatic Patients

### 3.1. Lipoprotein Receptors

Several years ago, Mommaas et al. noticed that there was an increased expression of LDL (low-density lipoprotein) receptors on the suprabasal cells in psoriatic skin and on the cell surface [25]. Sorokin et al. have reported lipoprotein-related receptor 1 (LRP1) to be increased in psoriatic patients [26]. Duvetorp et al. have investigated the role of lipoprotein-related receptors 5 and 6 (LRP5/6) in PSO. They have discovered that the expression of LRP5 and 6 in the psoriatic skin and blood is lower than in healthy subjects. Given that LRP5 and 6 are believed to act as anti-inflammatory particles and that after NB-UVB (narrowband-ultraviolet B) treatment, their expression has been shown to increase, they might be involved in psoriasis pathogenesis [27]. Another molecule that acts as a lipoprotein receptor is galectin-1 that has the ligand apolipoprotein A [28]. Galectin-1 is released from adipocytes. It has been investigated in psoriatic tissue, and it was revealed that it is not expressed in the affected epidermis, which might lead to disturbances in the proliferation of cells and keratinization [29]. CD36, which acts as a receptor for oxidized phospholipids, is upregulated in the epidermis in PSO [28,30].

Psoriasis, which is now perceived as systemic inflammation, often called ‘metaflammation’ to highlight the association with MS, is a factor contributing to increased lipoprotein oxidation in the form of oxidized modified lipoproteins (ox-LDL) [31]. Ox-LDL is known to stimulate inflammatory conditions and cholesterol accumulation in lysosomes, which results in cell death [1]. A study conducted by Shih et al. on mice with imiquimod-induced PSO has revealed that a high-cholesterol diet and ox-LDL lead to increased IL-23 expression in dendritic cells, which has a well-established place in PSO pathogenesis [32]. Ox-LDL promoted IL-23 through the lectin-type ox-LDL receptor 1 (LOX-1)-induced expression, which has been proved to be an important ox-LDL scavenging receptor [32,33]. Another proof for LOX-1 engagement in PSO is the fact of apremilast efficacy in PSO therapy. Apremilast is an approved antipsoriatic systemic agent which inhibits phosphodiesterase 4 (PDE4). It has been observed that apremilast inhibits the expression of LOX-1 [33]. Dey et al. have proved that sLOX-1 (soluble lectinlike oxidized low-density lipoprotein receptor-1) concentrations, which are found in serum after transformation of LOX-1 on the cell surface, are higher in psoriatic patients and are related to the severity of skin lesions [31]. Furthermore, these soluble receptors contribute to noncalcified coronary burden (assessed in computed tomography) rich in lipids in psoriatic patients [31].

Under normal conditions, HDL is meant to bind to scavenger receptor class B type 1 (SR-B1) on the surface of various cells, most importantly liver, which affects HDL-C serum concentrations [1]. This receptor has multiple beneficial functions. For instance, it is involved in cholesterol efflux and reverses cholesterol transport [1]. Increased expression of SR-B1 has been proved to inhibit atherosclerosis predisposition, whereas a decreased expression has been shown to promote atherosclerosis [1]. Since PSO is a disorder characterized by systemic inflammation, reverse cholesterol transport is less efficient, and the concentration of apolipoprotein A1 and the activity of enzymes associated with HDL are decreased. It affects HDL and inhibits its anti-inflammatory and antioxidative function [1].

### 3.2. Peroxisome Proliferator-Activated Receptors

Peroxisome proliferator-activated receptors (PPARs) are ligand-inducible transcription factors of the properties of nuclear hormone receptors which are involved in the metabolism of lipids and carbohydrates, as well as inflammatory and energy balance processes [34]. There are three types of PPARs that have been distinguished in humans: PPAR-α, PPAR-β/δ, and PPAR-γ. Since PPARs are involved in MS pathogenesis, their role in PSO, very closely related to such disorders, has already been investigated [34]. Apparently, in PSO, a reduced expression of PPAR-α and -γ has been observed, along with an increased expression of PPAR-β [35].

## 4. Aberrations in Lipid Metabolism in Psoriatic Patients

### 4.1. Lipids in Psoriatic Patients’ Skin

Skin plays an important role in lipid metabolism. Phospholipids, glucosylceramides, and sphingomyelin are enzymatically transformed in keratinocytes and then secreted to the intercellular matrix, where they are further processed into ceramides, free fatty acids (FFA), and cholesterol [35]. Ceramides are involved in skin-barrier maintenance, cell adhesion, epidermal cell differentiation, and stress-induced apoptosis. The total content of ceramides is similar between individuals who suffer from PSO and healthy persons, whereas in psoriatic lesions, there is an inadequate composition ratio of ceramides [1].

As for FFA, they are carboxylic acids with long aliphatic chains, not present in their ester-form FFAs, and are transported through plasma by a transport protein. The main source of fatty acids in animals is liver and adipose tissue [36]. They act as an energy supply for different tissues [36]. FFA content in psoriatic plaques is decreased [35].

The cholesterol in human tissues is engaged in cell membrane integrity maintenance and changes of their fluidity in response to changes in exposure to external factors. Cholesterol content in psoriatic skin is increased [1]. A study by Varshney et al. has revealed that signaling pathways via IL-17A lead to increased content of intracellular cholesterol, followed by suppression of cholesterol gene transcription. This contributes to the production of fatty acids in keratinocytes [37]. Psoriasis pathogenesis, which is already complex, is also associated with the abnormal function of the stratum corneum of the epidermis, along with its increased permeability and exfoliation due to improper enzymatic reactions in lipid metabolism [35]. Skin cholesterol was investigated as a marker of underlying vascular atherosclerosis, which is obviously closely related to PSO [38].

Enzymatic reactions during lipid metabolism are catalyzed by several enzymes. Serine palmitoyltransferase is involved in the first stages of ceramide synthesis [39]. Its expression in psoriatic lesions is decreased compared to healthy skin [40]. Sphingomyelinase is responsible for transforming sphingomyelin to ceramides. Its activity in the stratum corneum in PSO is decreased [1]. Ceramide synthases bind chains of fatty acids to sphingoid base, and elongases elongate fatty acid chains. One study has revealed that INFγ inhibits the expression of these two enzymes, suggesting that these processes might be the reason for ceramides content changes in psoriatic patients [41].

The expression of adipokines, which are secreted mainly by adipose tissue, is also abnormal in psoriatic skin. Leptin, which is involved in cell proliferation and inflammation in psoriatic skin, has been found to be overexpressed in psoriatic skin lesions in individuals with severe disease compared to those with a milder course or healthy persons [16]. Chemerin and progranulin are responsible for regulating inflammatory cell profiles in psoriatic skin [16]. Whereas progranulin expression in psoriatic skin has been proved to be elevated [16], in the case of chemerin, it is more complex [42]. In the early and active phase of skin involvement in psoriasis, the expression seems to be elevated, while in the chronic phase of this dermatosis, it decreases [42]. Resistin and visfatin contribute to the maintenance of the local inflammatory condition in psoriatic lesions. Their concentration has been proved to be elevated [16]. Adiponectin, vaspin, and omentin are regarded as anti-inflammatory adipokines, so their expression in psoriatic skin is decreased [16,43]. SERPINE1, which takes part in extracellular matrix degradation, is suspected to be overexpressed in the psoriatic tissue samples [16]. Zinc-α2-glycoprotein (ZAG) is engaged in epidermal cell differentiation and in psoriatic skin, particularly in the desquamation process [16]. It is not detected in the psoriatic plaque [44].

### 4.2. Lipids in Psoriatic Patients’ Blood

#### 4.2.1. Lipid Profile in Psoriatic Patients’ Blood

Lipid concentrations in psoriatic patients’ blood have been a subject of investigation for a long time. It is well established that psoriatic patients present an abnormal blood lipid profile, although different factors may affect this outcome, e.g., comorbidities, administered drugs, or diet [35]. A big meta-analysis of numerous studies investigating lipids in psoriasis concluded that total cholesterol level, along with LDL and VLDL, has been proved to be significantly higher in psoriatics than in control groups. This is similar to triglycerides and apolipoprotein B concentrations. On the other hand, HDL concentration is significantly decreased in psoriatic patients and, for apolipoprotein A, there seems to be no significant difference between patients and the control group [45]. One study has shown that psoriatic patients with dyslipidemia present increased concentrations of proinflammatory cytokine IL-6, which was also directly positively correlated with total cholesterol and LDL concentration, along with LDL/HDL ratio. Therefore, higher concentrations of this interleukin might indicate dyslipidemia occurrence in psoriatic patients [46]. Furthermore, soluble LOX-1 concentrations have been proved by Dey et al. to be increased in psoriatic patients and were associated with the severity of skin lesions [31]. PSO, with its chronic inflammation, is known to be a condition of elevated TNFα production, which may even be released from psoriatic plaques and act systemically [23]. It has been established that long-term elevation of TNFα affects lipid metabolism [1]. TNFα stimulates the production of small dense LDL and its oxidated form and inhibits the production of HDL [1].

As for FFA, they appear in human blood from hydrolysis of triglycerides in adipocytes or liver-derived LDL and are transported by albumins through plasma to destined tissues as energy supply [47]. Our team of dermatologists investigated the fatty acid profile in psoriatic patients. When it comes to the total concentration of fatty acids, it was not significantly different in psoriatic patients without comorbidities from the control group. However, the profile and concentration of particular fatty acids were significantly different between these groups. For instance, the proportion of monounsaturated fatty acids (MUFA) was increased, whereas, in terms of polyunsaturated fatty acids (PUFA), it was decreased. There was also an increased ratio of n-6/n-3 PUFA [48]. Research has shown that a fat-rich diet leads to an increase in FFA serum concentration and promotes the formation of saturated fatty acids (SFAs), which can affect immune processes and activate keratinocytes [1], hence the observations regarding obese persons (with positive calorie balance and inappropriate dietary habits) having more severe psoriatic skin lesions [49]. Furthermore, FFA affects lipoprotein metabolism since the increased content of FFA in the liver leads to intensified production of atherogenic LDL [49].

#### 4.2.2. Phospholipids

Phospholipids in psoriatic patients’ blood have already been studied. Research has shown that the total concentration of phospholipids, phosphatidylethanolamine, lecithin, linolenic acid, and docosatetraenoic and docosapentaenoic acids are decreased in patients with psoriasis [35]. In the case of palmitic and palmitoleic acids, data are inconsistent because some studies have revealed their increased concentrations, whereas no other significant difference was found in psoriatic patients compared to the control group [35]. A study by Zeng et al. revealed that the plasma concentrations of lysoglycerophosphoipids, such as lysophosphatidic acid (LPA) and lysophosphatidylcholine (LPC), were significantly increased in psoriatic patients. Among glycerophospholipids, phosphatidic acid (PA) concentration was also significantly increased, whereas phosphatidylcholine (PC) and phosphatidylinositol (PI) were significantly decreased in psoriatics [7].

#### 4.2.3. Adipokines in Psoriatic Patients’ Blood

**Figure 2:**
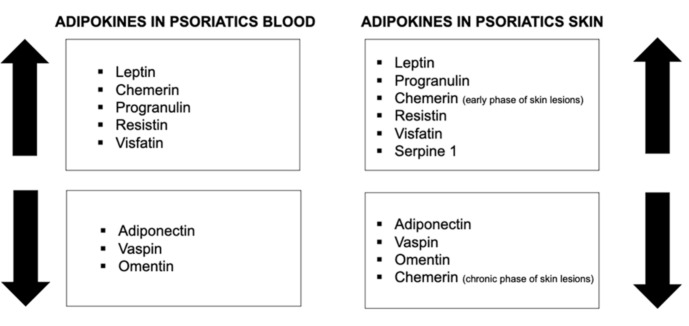
Adipokines in psoriatic patients’ skin and blood. 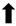 = increased level; 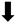 = decreased level.

The serum concentration of most adipokines is elevated in psoriatic patients (Figure 2). Leptin, chemerin, progranulin, resistin, and visfatin’s serum concentrations have been proved to be elevated [16,17]. On the other hand, adiponectin, vaspin, and omentin’s serum concentration are decreased in psoriatics [16,43,50]. However, there are still some inconsistencies between the published data, possibly for numerous reasons such as different ethnicity of the studied groups, interfering dietary or treatment modalities, or underlying comorbidities and methodological nuances.

#### 4.2.4. Fatty Acid-Binding Proteins in Psoriatic Patients

Fatty acid-binding proteins (FABPs) are a family of water-soluble proteins that are responsible for binding and transporting fatty acids to an appropriate destination in the human organism. There are 12 types of FABP distinguished, of which 10 isoforms are expressed in humans. Every FABP has its expression in one predominant cell or tissue [51]. In total, 5 out of 10 human FABP members have been proved to be abnormal in psoriasis.

FABP1 (liver FABP) and FABP4 (adipocyte FABP) have been proved by our team of dermatologists to be significantly elevated in the serum of psoriatic patients and might be predictive markers of clinical response to systemic treatment [52,53], whereas FABP3 (muscle and heart FABP) seems not to be significantly increased in this group of patients [54]. FABP2 (intestinal FABP), which is a marker of enterocyte damage, has been proved to be elevated in the plasma of psoriatic patients [55]. FABP5 (epidermal FABP) has been found to be involved in the metabolism of fatty acids, including epidermal cell differentiation [56]. Overexpression of FABP5 has been observed in psoriatic tissue and is even called psoriasis-associated FABP (PA-FABP) [56].

### 4.3. Lipids in Psoriatic Patients’ Biliary Metabolism

One study has analyzed the bile acid profile in psoriatic patients. The authors discovered that bile in such patients contains less conjugated primary bile acids, namely, glycocholate and glycochenodeoxycholate, and also secondary bile acids, namely, taurodeoxycholate, and glycodeoxycholate. Bile acids are involved in fat emulsion; therefore, their abnormal proportions lead to increased concentrations of cholesterol precursors in human plasma, namely, lathosterol and the oxidized derivative 7-β-hydroxycholesterol [26].

## 5. The influence of Lipid-Lowering Drugs on Psoriasis

Statins are synthetic inhibitors of 3-hydroxy-3-methylo-glutarylo-coenzyme A (HMG-CoA). They stimulate the expression of LDL receptors and, therefore, mainly decrease LDL concentrations [57]. They also mildly influence HDL and triglycerides concentrations, and they promote the stabilization of atherosclerotic plaque [57,58]. Statins are probably the most commonly used drugs in the treatment of dyslipidemia. Their influence on skin condition in psoriatics has already been investigated, and it has been shown that the severity of psoriatic lesions decreases after administration of statins in psoriatic patients [59].

Fibrates are agents which bind to nuclear PPARα, which activates them and leads to the promotion or inhibition of genes involved in lipoprotein metabolism. The result is a decrease in triglyceride, LDL, and VLDL concentrations and an increase in HDL [60]. The data on the impact of oral fibrates on psoriatic skin condition are sparse, but a single study suggests improvement of skin lesions during oral administration of clofibrate [61].

Glitazones are PPARγ agonists. Activation of such receptors leads to the transcription of genes involved in the metabolism of glucose and fatty acids. Glitazones decrease the blood concentration of FFA and glucose [34]. Some glitazones have been investigated in regard to skin lesion severity in psoriatics and revealed an improvement in skin condition after systemic administration. There have even been attempts to treat psoriasis with topical glitazones but without success [35].

PCSK9 inhibitors act by inhibiting PCSK9 (proprotein convertase subtilisin/kexin type 9), which prevents the binding of PCSK9 with LDL receptors on hepatocytes and the loss of such receptors, resulting in upregulation of their number and a decrease in LDL blood concentration [58]. Our team of dermatologists has investigated PCSK9 serum concentration in psoriatic patients and discovered that PCSK9 levels are significantly elevated in such patients and may become a marker of cardiometabolic disorder risk in PSO [62].

## 6. The Influence of Systemic Antipsoriatic Agents on Lipidemia

Among the basic systemic antipsoriatic agents, acitretin and cyclosporin A are known to cause hyperlipidemia as a side effect [8]. As for biological treatment, it has become the first-line treatment in the management of moderate-to-severe psoriasis [63]. The drugs act by selectively blocking cytokines involved in psoriatic inflammation, such as IL-17 or IL-23 and TNFα [63,64,65]. A study assessing lipid profile in psoriatics before and during anti-TNFα treatment resulted in a significant decrease in total and LDL cholesterol concentration, with a trend regarding the elevation of HDL and a decrease in triglyceride levels [66]. Since TNFα can induce the production of LDL and inhibit the production of HDL via decreased concentrations of lipoproteins, anti-TNFα agents promote beneficial changes in lipid profile [1]. Another study investigating the influence of secukinumab (an anti-IL-17A IgG1 antibody) on lipid parameters suggested a neutral effect [67]. Regarding ixekizumab (an anti-IL17A IgG4 antibody), there are inconsistent results, with one study suggesting that its influence on lipid parameters is neutral and another more recent study suggested it was beneficial [68,69]. The uncertain influence of anti-IL17 antibodies on lipid profile could be explained by inconsistent information regarding the nature of this cytokine. There have been findings reported in favor of both a protective and a pathogenic role of this cytokine [70]. Varshney et al. showed that during IL-17A signaling, total cholesterol concentration in psoriatic epidermis was elevated. Moreover, they suggested that such increased levels of intracellular cholesterol may subsequently influence the cholesterol concentration in the blood, which could lead to the low blood concentration of HDL and elevated concentration of LDL in patients with psoriasis [37]. It has also been established by Manti et al. that serum IL-17 and Il-23 are positively correlated with the total and LDL cholesterol concentration and negatively correlated with HDL concentration at the same time [71]. Anti-17A antibodies bind to IL-17 and neutralize it; therefore, they might influence lipid profile. As for ustekinumab, which is a human IgG1 anti-IL-12/23 antibody, in one randomized, double-blind, placebo-controlled crossover study on psoriatics, after 12 weeks of therapy with this agent, there was significantly elevated LDL concentration and LDL-particle number. However, significantly, these changes were transient and were not present at 52 weeks of therapy [72]. Biologic therapy for psoriasis has also been proved to have a beneficial influence on the modification of the lipid-rich necrotic core, which is a high-risk coronary plaque feature [73].

## 7. Conclusions

Lipids play an indisputable role in psoriasis. Aberrations of lipid expression and metabolism, as well as lipids’ transporting proteins and receptors, are frequently present in psoriatic patients. Psoriatic patients suffer more often from hyperlipidemia and are prone to develop MS, atherosclerosis, and thus cardiovascular disorders. Moreover, different drugs used in psoriasis therapy have a positive or negative impact on lipid profile; therefore, they must be chosen carefully. On the other hand, antihyperlipidemic drugs may benefit psoriatic patients, not only in the treatment of dyslipidemia but also in improving skin conditions.

## 8. Materials and Methods

The research was conducted using the PubMed database. The keywords used for the search were different combinations of ‘psoriasis’ and ‘lipids’ or ‘fatty acids’ or ‘phospholipids’ or ‘metabolic syndrome’ or ‘obesity’ or ‘lipid profile’ or ‘lipoproteins’ or ‘atherosclerosis’ or ‘bile’ or ‘fatty-acid binding proteins’ or ‘peroxisome proliferator-activated receptors’ or ‘adipokines’.

## Data Availability

No new data generated.

## Abbreviations

BMI	body mass index
CD	cluster antigen
CMDs	cardiometabolic disorders
DM	diabetes mellitus
FABPs	fatty acid-binding proteins
FFA	free fatty acids
HDL	high-density lipoprotein
HLA	human leukocyte antigen
HMG-CoA	3-hydroxy-3-methylo-glutarylo-coenzyme A
IL	interleukin
INFγ	interferon gamma
LDL	low-density lipoprotein
LRP	lipoprotein-related receptors
LOX-1	lectin-type ox-LDL receptor
LPA	lysophosphatidic acid
LPC	lysophosphatidylcholine
MS	metabolic syndrome
MUFA	monounsaturated fatty acids
NAFLD	nonalcoholic fatty liver disease
NB-UVB	Narrowband—ultraviolet B
ox-LDL	oxidized modified low-density lipoproteins
PA	phosphatidic acid
PASI	psoriasis activity and severity index
PCSK9	proprotein convertase subtilisin/kexin type 9
PDE4	phosphodiesterase 4
PI	phosphatidylinositol
PPARs	peroxisome proliferator-activated receptors
PSO	psoriasis
PUFA	polyunsaturated fatty acids
SFAs	saturated fatty acids
sLOX-1	soluble lectinlike oxidized low-density lipoprotein receptor-1
SR-B1	scavenger receptor class B type 1
TNFα	tumor necrosis factor-alpha
VLDL	very-low-density lipoprotein
ZAG	zinc-α2-glycoprotein
